# PGC‐1α regulates the interplay between oxidative stress, senescence and autophagy in the ageing retina important in age‐related macular degeneration

**DOI:** 10.1111/jcmm.18051

**Published:** 2024-04-03

**Authors:** Iswariyaraja Sridevi Gurubaran, Cezary Watala, Joanna Kostanek, Joanna Szczepanska, Elzbieta Pawlowska, Kai Kaarniranta, Janusz Blasiak

**Affiliations:** ^1^ Department of Ophthalmology University of Eastern Finland Kuopio Finland; ^2^ Department of Haemostatic Disorders Medical University of Lodz Lodz Poland; ^3^ Department of Pediatric Dentistry Medical University of Lodz Lodz Poland; ^4^ Department of Ophthalmology Kuopio University Hospital Kuopio Finland; ^5^ Faculty of Medicine, Collegium Medicum Mazovian Academy in Plock Plock 09‐402 Poland

**Keywords:** age‐related macular degeneration, ageing retina, AMD, autophagy, cellular senescence, oxidative stress, peroxisome proliferator‐activated receptor gamma coactivator 1‐alpha, PGC‐1α

## Abstract

We previously showed that mice with knockout in the peroxisome proliferator‐activated receptor gamma coactivator 1‐alpha *(PPARGC1A*) gene encoding the PGC‐1α protein, and nuclear factor erythroid 2 like 2 (*NFE2L2*) gene, exhibited some features of the age‐related macular degeneration (AMD) phenotype. To further explore the mechanism behind the involvement of PGC‐1α in AMD pathogenesis we used young (3‐month) and old (12‐month) mice with knockout in the *PPARGC1A* gene and age‐matched wild‐type (WT) animals. An immunohistochemical analysis showed age‐dependent different expression of markers of oxidative stress defence, senescence and autophagy in the retinal pigment epithelium of KO animals as compared with their WT counterparts. Multivariate inference testing showed that senescence and autophagy proteins had the greatest impact on the discrimination between KO and WT 3‐month animals, but proteins of antioxidant defence also contributed to that discrimination. A bioinformatic analysis showed that PGC‐1α might coordinate the interplay between genes encoding proteins involved in antioxidant defence, senescence and autophagy in the ageing retina. These data support importance of PGC‐1α in AMD pathogenesis and confirm the utility of mice with PGC‐1α knockout as an animal model to study AMD pathogenesis.

## 1 INTRODUCTION

Age‐related macular degeneration (AMD) is an eye disease affecting ageing populations that may lead to legal blindness and sight loss with ageing and oxidative stress consistently reported as main AMD risk factors.^1^ AMD affects the retinal pigment epithelium (RPE) cells with subsequent degeneration of photoreceptors, resulting in visual disturbances and ultimately—vision loss. AMD can be clinically divided into two distinct forms: dry and wet. The former is incurable but targeting the vascular endothelial growth factor A (VEGFA) with its antibodies and inhibitors of their receptors, may stop the progression of wet AMD and protect against sight loss.^2^ Effective treatment of AMD is impeded by a poor knowledge of the disease pathogenesis, which is, at least in part, underlined by a restricted accessibility of the human retina for research and limited adequacy of cellular and animal models to mimic human AMD. We established an animal AMD model with mice carrying mutations in the nuclear factor erythroid 2 like 2 (*NFE2L2*) and peroxisome proliferator‐activated receptor gamma coactivator 1‐alpha (*PPARGC1A*) genes (dKO mice), whose phenotype resembled dry AMD.^3^ These transgenic animals showed a significant age‐dependent RPE degeneration, an increase in the oxidative stress and endoplasmic reticulum markers, 4‐HNE (4‐hydroxynonenal) and GRP78 (glucose‐regulated protein 78) and damaged mitochondria. Mice bearing mutations in the *NFE2L2* and *PPARGC1A* genes displayed changes in protein ubiquitination and autophagy markers. These mice showed morphological defects in photoreceptors that were associated with vision loss. Therefore, the expression of *NFE2L2* and *PPARGC1A* genes may play a role in AMD pathogenesis. In a subsequent work we showed that *NFE2L2/PPARGC1A* dKO mice showed some alterations in inflammatory pathways that are important in AMD pathogenesis.^3^ We also showed that the dKO mice displayed an alternated expression of epithelial‐mesenchymal transition (EMT) transcription factors.^4^ Also, increased immunoreactivity of senescence markers p16, basic helix–loop–helix family member E40 (DEC1) and high mobility group box 1 (HMGB1) were shown, suggesting that EMT and senescence might intersect in the retina of dKO mice, contributing to an AMD‐like phenotype. Both EMT and cellular senescence may be stimulated by oxidative stress, which may be potentiated by the absence of *NFE2L2* and *PGC‐1α* genes, important in antioxidant defence.^5^

*NFE2L2* and *PPARGC1A* may be involved in AMD pathogenesis in several pathways and their combined effects may be related to the mechanism of the action of any single gene. Golestaneh and her coworkers showed that PGC‐1α was important in AMD pathogenesis, mainly through its involvement in mitochondrial homeostasis, autophagy and metabolic pathways as well as an interaction with the anti‐ageing hormone, Klotho.^6^, ^7^, ^8^, ^9^ They used mice with knockout in the *PPARGC1A* gene, RPE from native AMD eyes and human RPE cells derived from induced pluripotent stem cells obtained from skin of AMD donors. They showed that *PPARGCA1*(+/−) mice expressing lower levels of PGC‐1α than their WT counterparts displayed an AMD‐like phenotype when administrated with high‐fat diet, a consistently reported AMD risk factor.^7^ Also, Saint‐Geniez et al. using *PPARGC1A* KO mice showed an import role of PGC‐1α in retinal angiogenesis, a key event in the pathogenesis of wet AMD.^10^ They also showed that induction of PGC‐1α promoted EMT and metabolism in RPE cells and protected them against oxidative damage.^11^, ^12^

PGC‐1α is a co‐activator of nuclear receptors and transcription factors involved in the regulation of many aspects of cellular homeostasis, including the reaction to oxidative stress and ageing.^13^, ^14^ PGC‐1α belongs to the peroxisome proliferator‐activated receptor gamma coactivator‐1 (PGC‐1) family, which consists of PGC‐1α, PGC‐1β and PRC (PGC‐1‐related coactivator).^15^ Apart from oxidative stress, PGC‐1α is involved in the regulation of cellular senescence and autophagy, processes that play an important role in AMD pathogenesis.^16^, ^17^, ^18^ On the contrary, modification of autophagy inducers may regulate PGC‐1α activity.^19^ Generally, PGC‐1α is expressed at relatively high levels in the mouse retina, so mice are a good subject to study PGC‐1α.^20^ Aging is per definition a crucial AMD risk factor and PGC‐1α plays an important role in ageing, mainly through its involvement in mitochondrial energy metabolism.^13^ This justifies studies on the role of PGC‐1α in the ageing retina in AMD pathogenesis.

Inspired by these and our own results we further explored the role of PGC‐1α in the pathways important in AMD pathogenesis: antioxidant defence, senescence and autophagy in young and old mice.^11^ In the present work, we verified that PGC‐1α played a role in the interplay between oxidative stress, senescence and autophagy in the ageing retina. To do so, we immunohistochemically determined the expression of genes involved in oxidative stress defence, senescence and autophagy in transgenic mice carrying knock out in the *PPARGC1A* gene and WT animals. In addition, we performed a bioinformatic analysis of interactions between the genes, whose expression we determined.

## 2 MATERIALS AND METHODS

### 2.1 Reagents

Tris‐based antigen unmasking solution was purchased in Vector Laboratories Inc. (Burlingame, CA, USA), DAPI (4′,6‐diamidino‐2‐phenylindole dihydrochloride) was obtained from Sigma‐Aldrich (St. Louis, MO, USA). Primary and secondary antibodies and their suppliers were listed in further text. Mowiol mounting media was made in our laboratory.

### 2.2 Animals

All animal experiments were performed according to the protocols that agreed with the ARVO Statement for the Use of Animals in Ophthalmic and Vision Research and approved by the Project Authorization Board of Regional Administrative Agency for Southern Finland (ESAVI/8893/04.10.07/2014). The animals were maintained in a 12/12 h light–dark cycle at constant temperature 22 ± 1°C and had a free access to drinking water and standard pellet chow.

Transgenic mice with constitutive knockout in the *PPARGC1A* gene (PGC‐1α KO mice) were originally provided by Dr. Bruce Spiegelman of Dana Faber Cancer Institute, Philadelphia, PA, USA and generated from the C57BL/6J strain as described elsewhere.^21^ The animals were genotyped as described previously.^22^ The 3‐ or 12‐month‐old mice were sacrificed, and their eyes were collected immediately, enucleated and fixed in 2% paraformaldehyde for 2 h.

### 2.3 Immunohistochemistry

Gene expression in the mice RPE was evaluated by immunohistochemistry. We studied the expression of the following genes coding for proteins involved in the cellular response to oxidative stress: *APEX1* (apurinic/apyrimidinic endodeoxyribonuclease 1, APE1), *OGG1* (8‐oxoguanine DNA glycosylase), *PDIA2* (protein disulfide isomerase family A member 2), *TXN* (thioredoxin), *SOD1* (superoxide dismutase 1) and *H2AX* (H2A.X variant histone, H2AX phosphorylated at Ser139). The genes encoding proteins involved in cellular senescence were: *TP53* (tumour protein p53), *HMGB1* (high mobility group box 1), *CDKN1A* (cyclin dependent kinase inhibitor 1A, p21), *CDKN2A* (cyclin dependent kinase inhibitor 2A, p16INK4a, p16) and *BHLHE40* (basic helix–loop–helix family member E40, DEC1). The genes involved in macroautophagy and mitophagy were *BECN1* (BECLIN1), *LAMP2* (lysosomal associated membrane protein 2), *MAP1LC3B* (microtubule associated protein 1 light chain 3 beta, LC3B), *SQSTM1* (sequestosome 1, p62), *UBB* (ubiquitin B), *PINK1* (PTEN induced kinase 1) and *PRKN* (parkin RBR E3 ubiquitin protein ligase, PRK8).

Tissues sections were deparaffinized using xylene, rehydrated, washed and pretreated with Tris‐based antigen unmasking solution for 7.5 min at 90°C. The sections were encircled with a PAP pen and quenched with 0.1 M glycine in PBS for 10 min prior to a 0.1% Triton‐X wash for 10 min before proceeding with blocking for 30 min. For single staining, quenched slides were incubated with 20% goat serum for 30 min before adding a primary antibody and incubated 24 h at 4°C. Then, the slides were washed with TBS and incubated with the secondary antibody for 3 h at room temperature. For double staining (PINK1/PRKN), the first primary antibody (PINK1) was added followed by its secondary antibody and then the second primary antibody (PRKN) was added, followed by its secondary antibody. The secondary antibodies were goat anti‐rabbit Alexa Fluor 488 and 594 (A11034/A11037) and goat anti‐mouse Alexa Fluor 594 (A11032) (Thermo Fisher Scientific, Waltham, MA, USA) diluted at 1:500 in TBS.

### 2.4 Data acquiring

The slides were mounted using the Mowiol mounting media and stored in the dark at room temperature. The stained sections were examined with a confocal microscope (Carl Zeiss AX10 Imager A2, Carl Zeiss Microscopy, Jena, Germany) using a 63× (NA:1.42. Plan Apochromat) oil (Zeiss Immersol™) immersion objective. At least nine repetitive images were taken from each section for all markers. Images were colour enhanced using Adobe photoshop for visual representation. All the captured images were converted into 8‐bit and processed using ImageJ v1.53t (https://imagej.nih.gov/ij/). The background was subtracted using a default rolling ball radius method. Regions of interest (ROI) were drawn over RPE cell layer followed by colocalization analysis using 2 channel spots colocalization analyser ComDet v0.5.5 (https://imagej.net/plugins/spots‐colocalization‐comdet), an ImageJ plugin. The total number of puncta from each colour (channel) was calculated and the corresponding correlations of colocalization were measured. The mean grey value was used to quantify optical changes seen in images.

### 2.5 Data analysis

Six mice (3 WT and 3 KO) aged 3 months and 8 mice (4 WT and 4 KO) aged 12 months were used in this study. Three sections from each mouse were stained for each antibody and 4–6 regions of interest (ROIs) were randomly analysed in each section. The actual range of numbers of readings was indicated in each figure legend. Data were presented as the BCA bootstrap‐boosted mean + SD or median with interquartile range (lower quartile [25%] to upper quartile [75%]), depending on data distribution. Data normality and variance homogeneity of the acquired data were verified using Shapiro–Wilk's and Levene's test, respectively. The data complying with the assumptions of normal distribution and homogeneity of variances were analysed with Student's *t*‐test and one‐way or two‐way anova (either block or main component model or the model with interactions, both in the version with replicates), while for the remaining data with a Mann–Whitney rank sum *U*‐test and Kruskal‐Wallis test. In order to assess the significance of differences between particular samples, we used the post‐hoc multiple comparisons tests (the least significant difference test [LSD] or the Bonferroni's correction for multiple comparisons). The resampling bootstrap technique (10,000 iterations) was used to minimize the risk that the revealed differences were observed by pure chance. Statistical analyses were performed using Statistica v.13 (Dell Inc., Tulsa, OH, USA), GraphPad Prism for Windows ver. 9.5 (GraphPad Software, Boston, MA, USA), Resampling Stats Add‐In for Excel v.4 (The Institute for Statistics Education, An Elder Research Company, Arlington, VA, USA) and R Package Software v. 4.4, in which we used the algorithm for data resampling in various employed analyses, written by one of the authors (J.K.).

Multivariate inference testing was accomplished in three alternative approaches. First, we used linear discriminant analysis (LDA) to verify which proteins in three sets (antioxidant defence, senescence and autophagy) contributed mostly to the best discrimination between KO and WT mice. Second, we employed manova and T2 Hotelling test to verify whether the overall sets of proteins studied differed between KO and WT animals. Third, we transformed real values in each variable into normal scores according to the van der Waerden formula.^23^ Next, we calculated cumulative scores (CSs) by summing up the relevant normal scores across any given set of proteins (antioxidant defence, senescence or autophagy), or alternatively across all variables. The resultant CSs were further used for the inference analyses.

In bioinformatic analysis, gene expression data were normalized with interquartile range method. Principal component analysis (PCA) was performed with FactoMineR and factoextra packages in the R 4.2.2 for Windows software (R Development Core Team, Vienna, Austria). Interaction networks were constructed using the StringApp for Cytoscape, an open source bioinformatic software (version 3.9.1). A score exceeding 0.4 was set as a threshold for considering an interaction.

## 3 RESULTS

First, we validated the main experimental method we used, immunohistochemistry, to verify the lack of PGC‐1α in the KO animals. Although a background signal was relatively strong, we confirmed the lack of the expression of the *PPARGCA1* gene in the PGC‐1α KO mice (results not shown). Moreover, we validated immunohistochemistry as a suitable method in our experimental conditions.

### 3.1 Antioxidant defence

**FIGURE 1:**
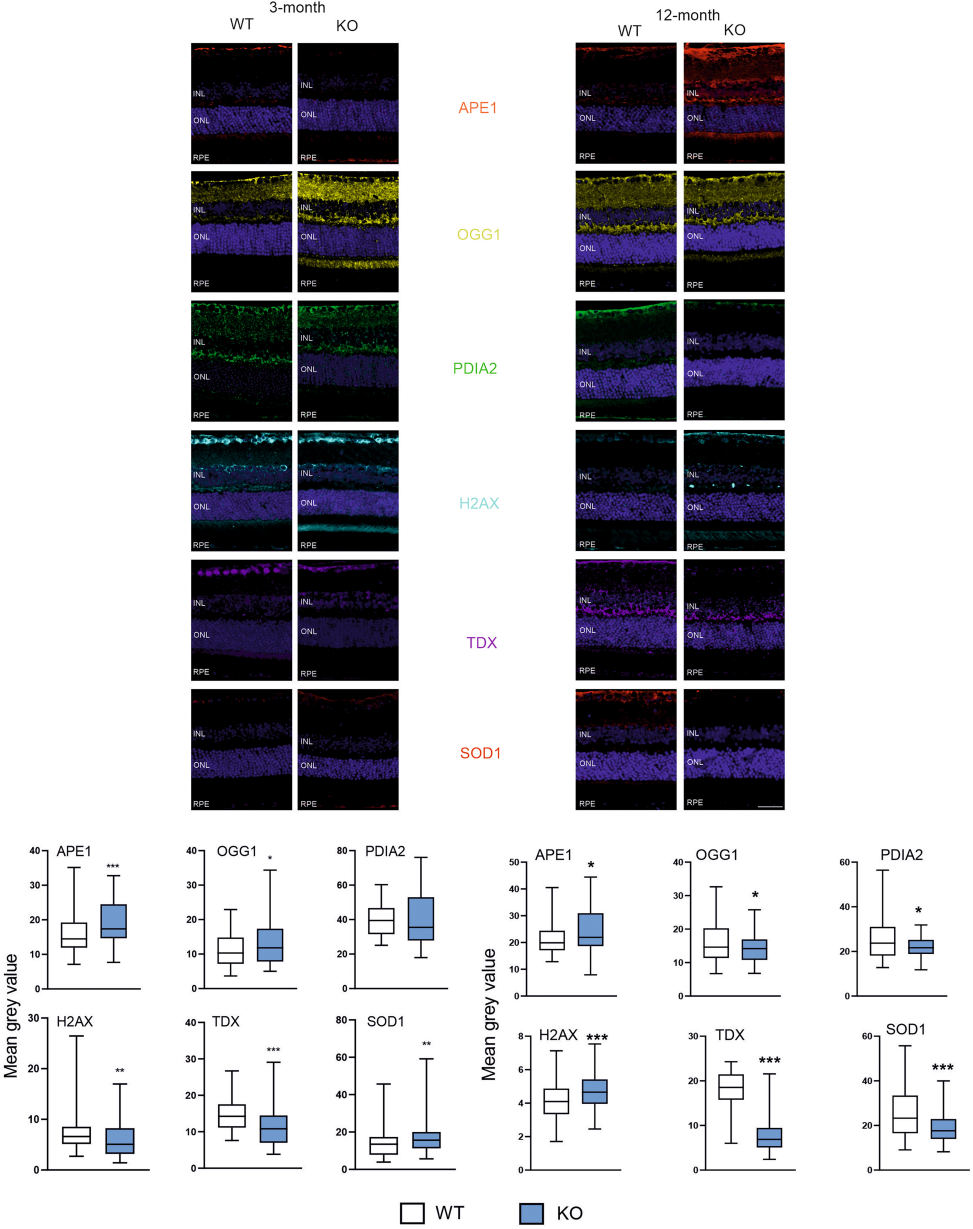
Peroxisome proliferator‐activated receptor gamma coactivator 1‐alpha (PGC‐1α) influences the expression of markers of antioxidant defence in the retinal pigment epithelium (RPE) of young and old mice. Confocal microscopy whole retina images of the immunoreactivity of the markers of antioxidant defence: APE1 (apurinic/apyrimidinic endodeoxyribonuclease 1), OGG1 (8‐oxoguanine DNA glycosylase), PDIA2 (protein disulfide isomerase family A member 2), TDX (thioredoxin), SOD1 (superoxide dismutase 1) and H2AX (H2A.X variant histone H2AX phosphorylated at Ser139) in RPE of wild‐type (WT) and mice with the global knockout in the *PPARGC1* gene encoding the PGC‐1α protein (KO) aged 3 or 12 months. Puncta specific to the label of the primary antibody to each marker have the same colour as its abbreviation displayed between image panels. DAPI was used to stain the nuclei of RPE cells (blue). ONL, outer nuclear level; INL, inner nuclear level. Scale bar: 50 μm (upper panels). Mean grey value for the markers of antioxidant defence determined in WT and KO mice. Median ± lower and upper quartile, error bars represent minimum and maximal values; the number of readings *n* = 76–175; *p < 0.05, **p < 0.01, ***p < 0.001 as compared with WT animals (lower panels).

Figure 1 presents results of immunohistochemical determination of the expression of markers of antioxidant defence in young (3‐month) and old (12‐month) mice with knockout in the gene encoding PGC‐1α. APE1, OGG1 and SOD1 increased in 3‐month KO animals, whereas H2AX (pSer139) and TDX—decreased. No changes were observed in PDIA2. 12‐month KO mice displayed higher expression of APE1 and H2AX (pSer239), while OGG1, PDIA2, TDX and SOD1 in KO animals decreased as compared with their WT counterparts.

As the results of the expression of all markers were influenced by two variables, the expression of the *PGCRCA1* gene (KO vs. WT) and animal age (3 vs. 12‐month), we investigated the interaction between these two factors in their effect on the markers expression. We observed that the expression of APE1, PDIA2, TDX were affected by both age and the presence/absence of PGC‐1α (*p* < 0.05).

### 3.2 Senescence

**FIGURE 2:**
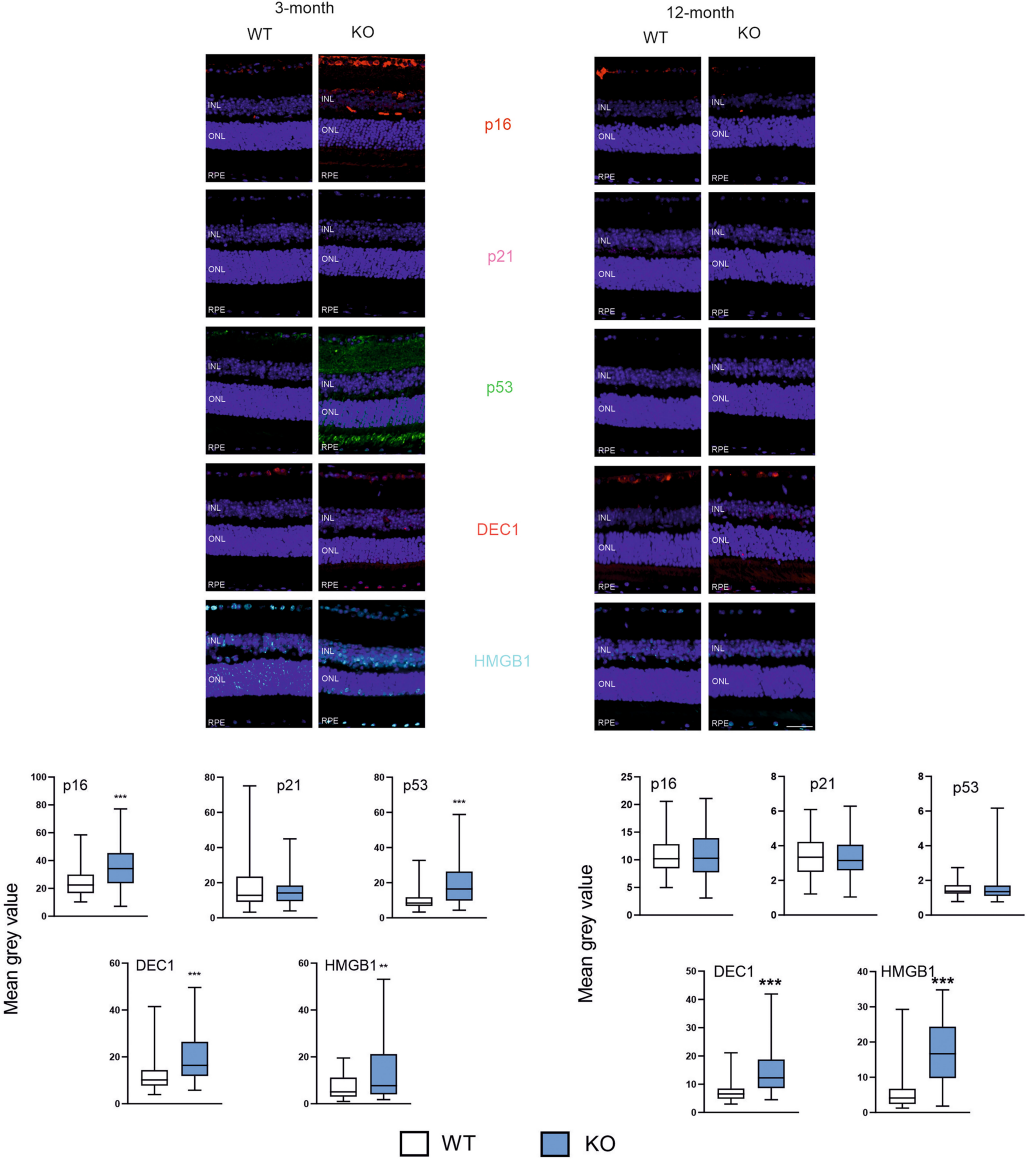
Peroxisome proliferator‐activated receptor gamma coactivator 1‐alpha (PGC‐1α) influences the expression of markers of senescence in the retinal pigment epithelium (RPE) of young and old mice. Confocal microscopy whole retina images of the immunoreactivity of the markers of senescence: p16 (cyclin dependent kinase inhibitor 2A), p21 (cyclin dependent kinase inhibitor 1A), p53 (tumour protein p53), DEC1 (basic helix–loop–helix family member E40) and HMGB1 (high mobility group box 1) in RPE of wild‐type (WT) and mice with the global knockout in the peroxisome proliferator‐activated receptor gamma coactivator 1‐alpha gene (*PPARGC1A*) encoding the PGC‐1α protein (KO). Puncta specific to the label of the primary antibody to each marker have the same colour as their abbreviations between images. DAPI was used to stain the nuclei of RPE cells (blue). ONL, outer nuclear level; INL, inner nuclear level. Scale bar: 50 μm (upper panels). Mean grey value for the markers of antioxidant defence determined in WT and KO mice. Median ± lower and upper quartile, error bars present minimum and maximal values; the number of readings *n* = 51–105; **p < 0.01, ***p < 0.001 as compared with WT animals (lower panels).

Three‐month KO animals displayed an increased expression of p16, p53, DEC1 and HMGB1, while p21 did not change (Figure 2). In 12‐month mice, DEC1 and HMGB1 increased in KO as compared with WT animals. No changes were observed in the expression of p16, p21 and p53 markers in KO and WT animals.

The expressions of p16, p53 and DEC1 were affected by both time and the presence/absence of PGC‐1α and these factors interacted with each other (*p* < 0.001).

### 3.3 Autophagy

**FIGURE 3:**
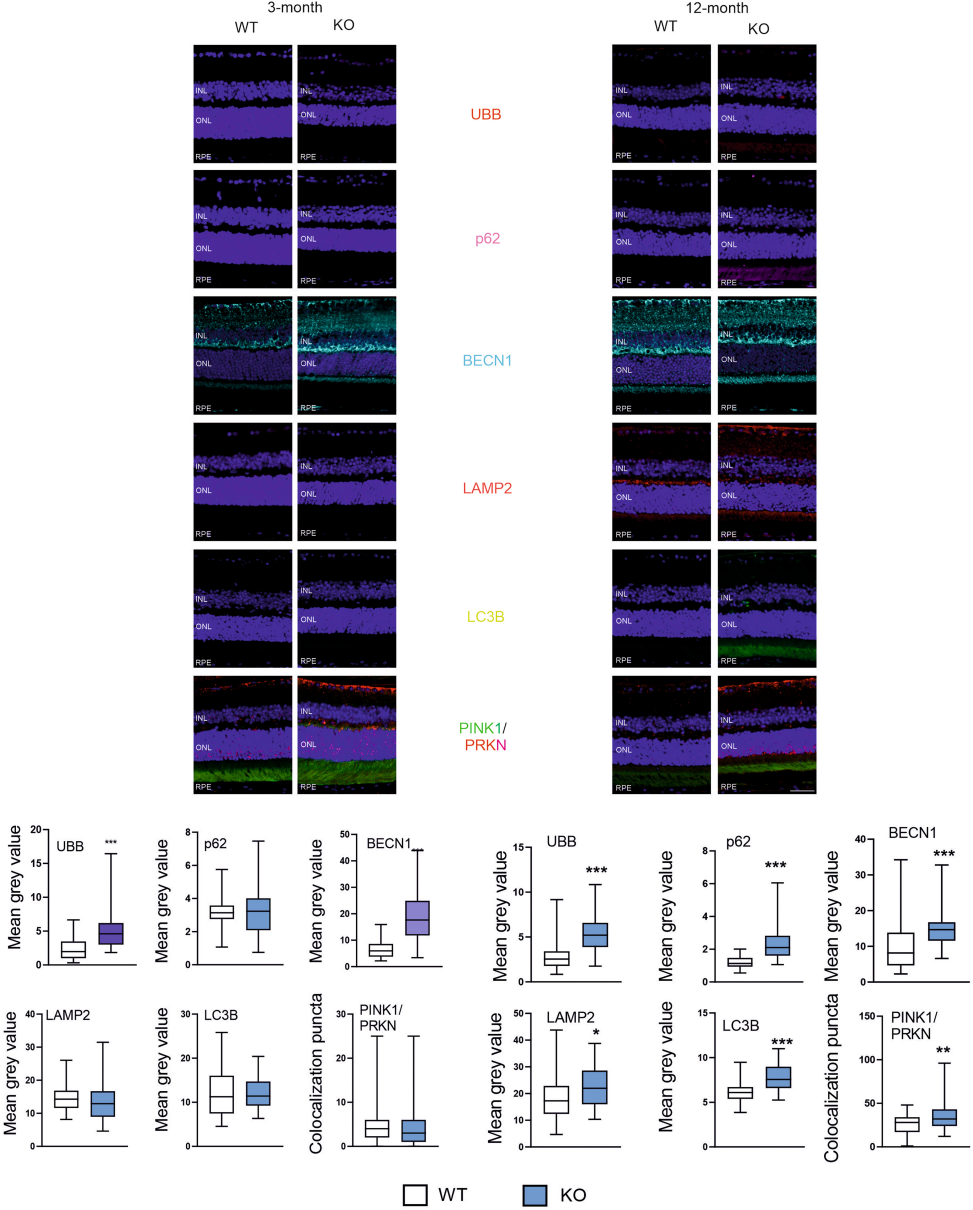
Peroxisome proliferator‐activated receptor gamma coactivator 1‐alpha (PGC‐1α) influences the expression of markers of autophagy in the retinal pigment epithelium (RPE) of young and old mice. Confocal microscopy whole retina images of the immunoreactivity of the markers of autophagy: UBB (ubiquitin), p62 (sequestosome 1), BECN1 (BECLIN1), LAMP2 (lysosomal associated membrane protein 2), MAP1LC3B (microtubule associated protein 1 light chain 3 beta, LC3B), p62 (SQSTM1, sequestosome 1), PINK1 (PTEN induced kinase 1) and PRKN (parkin RBR E3 ubiquitin protein ligase) in RPE of wild‐type (WT) and mice with the global knockout in the *PPARGC1A* gene encoding the PGC‐1α protein (KO). Puncta specific to the label of the primary antibody to each marker have the same colour as their abbreviations placed between image panels. DAPI was used to stain the nuclei of RPE cells (blue). Arrows in the PINK/PRKN image show clusters of colocalization of both markers. ONL, outer nuclear level; INL, inner nuclear level. Scale bar: 50 μm (upper panels). Mean grey value for the markers of antioxidant defence determined in WT and KO mice. Median ± lower and upper quartile, error bars present minimum and maximal values; the number of readings *n* = 43–81; *p < 0.05, **p < 0.01, ***p < 0.001 as compared with WT animals (lower panels).

Ubiquitin and BECN1 showed an increased expression in 3‐month KO animals as compared with their WT counterparts, while p62, LAMP‐2 and LC3B were not changed (Figure 3). Twelve‐month KO mice showed an increased expression of all six autophagic markers as compared with the WT animals. The expressions of p62, LAMP2 and the PINK1/PRKN ratio were affected by both age and PGC‐1a and there was an interaction between them (*p* < 0.05 for all factors).

### 3.4 Multivariate interference testing

To determine contribution of a specific group of proteins (antioxidant defence, senescence and autophagy) and a specific protein within each group to the discrimination between PGC‐1α KO and WT cells a multivariate interference testing was performed.^24^

We observed a stronger discrimination between PGC‐1α KO and WT mice for 12‐month than 3‐month animals as we noted a greater number of markers contributing to differences between the KO and WT in old than in young animals and these differences were greater in the former than the latter (Table 1).

**TABLE 1:** Markers of antioxidant defence, senescence and autophagy most significantly contributing to the discrimination between gene expression in PGC‐1α KO and WT animals.

Group	3‐month						12‐month					
	Protein	*p* (less than)					Protein	*p* (less than)				
		LDA		manova	Student *t*	T^2^		LDA		manova	Student *t*	T^2^
		*P*_variables_	*P*_MD_					*P*_variables_	*P*_MD_			
Antioxidant defence	APE1	0.05	0.05	0.05	0.05	0.001	APE1	0.001	0.001	0.001	0.001	0.001
	TDX	0.04			0.05		TDX	0.01			0.001	
							H2AX	0.01			0.05	
Senescence	HMGB1	0.01	0.001	0.05	0.05	0.001	HMGB1	0.001	0.001	0.001	0.001	0.001
	DEC1	0.01			0.05		DEC1	0.05			0.01	
	p53	0.05			0.001							0.001
Autophagy	BECN1	0.01	0.001	0.001	0.001	0.001	p62	0.05	0.001	0.001	0.01	
	UBB	0.05			0.01		BECN1	0.05			0.01	
							LC3B	0.01			0.001	

Abbreviations: LDA, linear discriminant analysis; MONOVA, refers to the Wilks' significance of differences between the groups based on multivariate inference testing; *P*_MD,_ significance of Mahalanobis' distance; *P*_variables_, significance of variables contributing to the best discrimination between groups; Student t, significance of differences between individual variables; T^2^—Hotelling, refers to the significance of multivariate comparison between two groups.

### 3.5 Bioinformatic analysis

To assess the role of PGC‐1α and ageing in mutual interactions between genes and proteins of antioxidant defence, senescence and autophagy, a bioinformatic analysis was performed.

**FIGURE 4:**
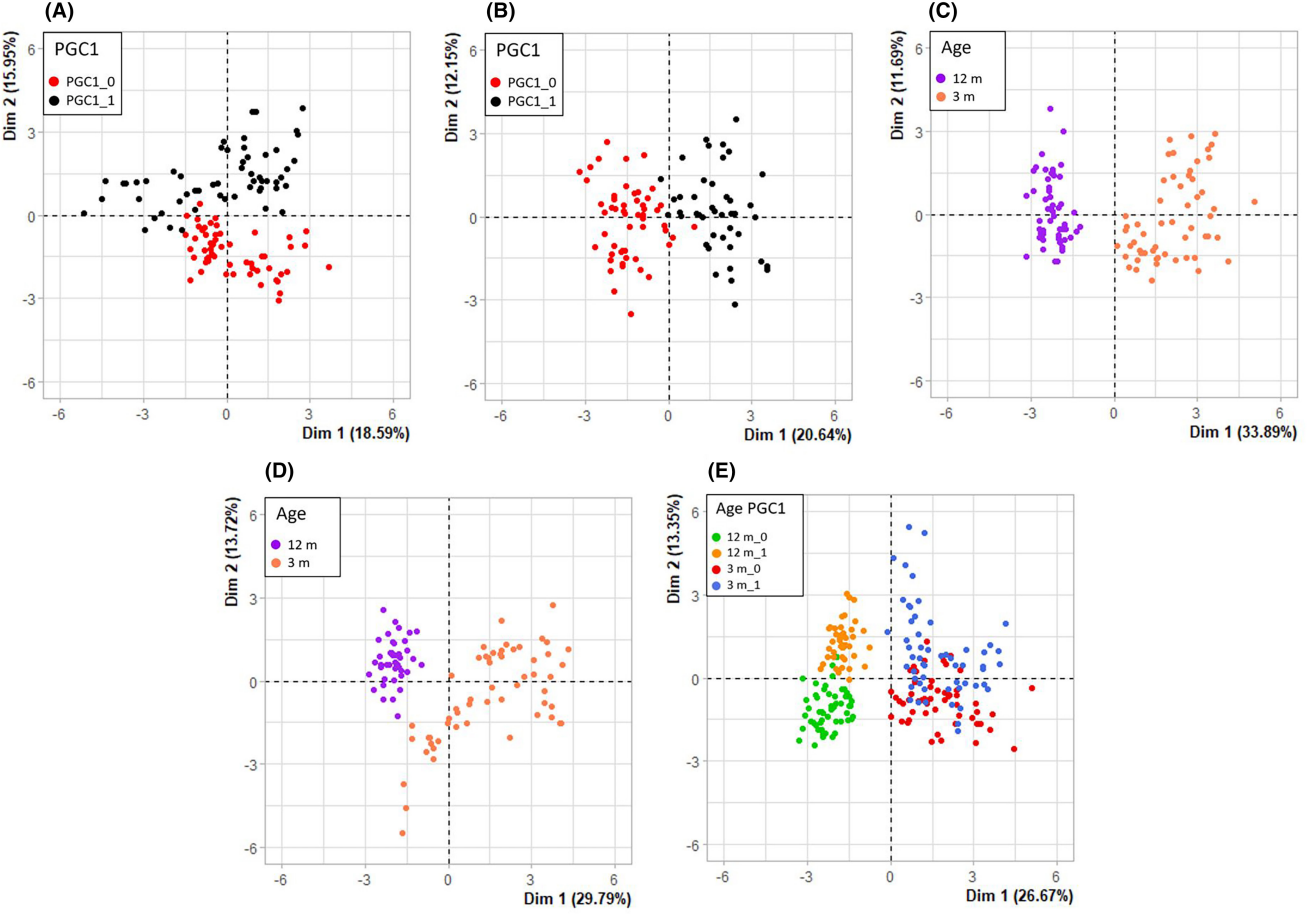
Principal component analysis (PCA) of the expression of genes involved in antioxidant defence, senescence and autophagy in the ageing retina of mice with knockout in the peroxisome proliferator‐activated receptor gamma coactivator 1‐alpha (*PPARGC1A*) gene encoding PGC‐1α gene and wild‐type animals. Each dot represents the expression of a single gene. PCA was performed for 3‐month (A) and 12‐month (B) mice, PGC‐1α knockout (C) and wild‐type (D) mice or data for all kinds of mice (E). PGC‐1α is abbreviated to PGC1, its expression in WT animals is designated by 1 and its lack—by 0.

Although our work was far from typical high‐throughput analysis, the number of analysed genes (17), variants of age (2) and PGC‐1α expression (2) made it necessary to reduce dimensions, which can be done by PCA.^25^ Figure 4 presents PCA with dimensional reduction on PGC‐1α expression (A, B) and age (C, D) as well as data without dimensional reduction. It can be seen that PGC‐1α expression moderately separated the data (A, B), but when the separation was done by age, the data were strongly split into two non‐overlapping clusters (C, D), which was confirmed by PCA for all data (E).

**FIGURE 5:**
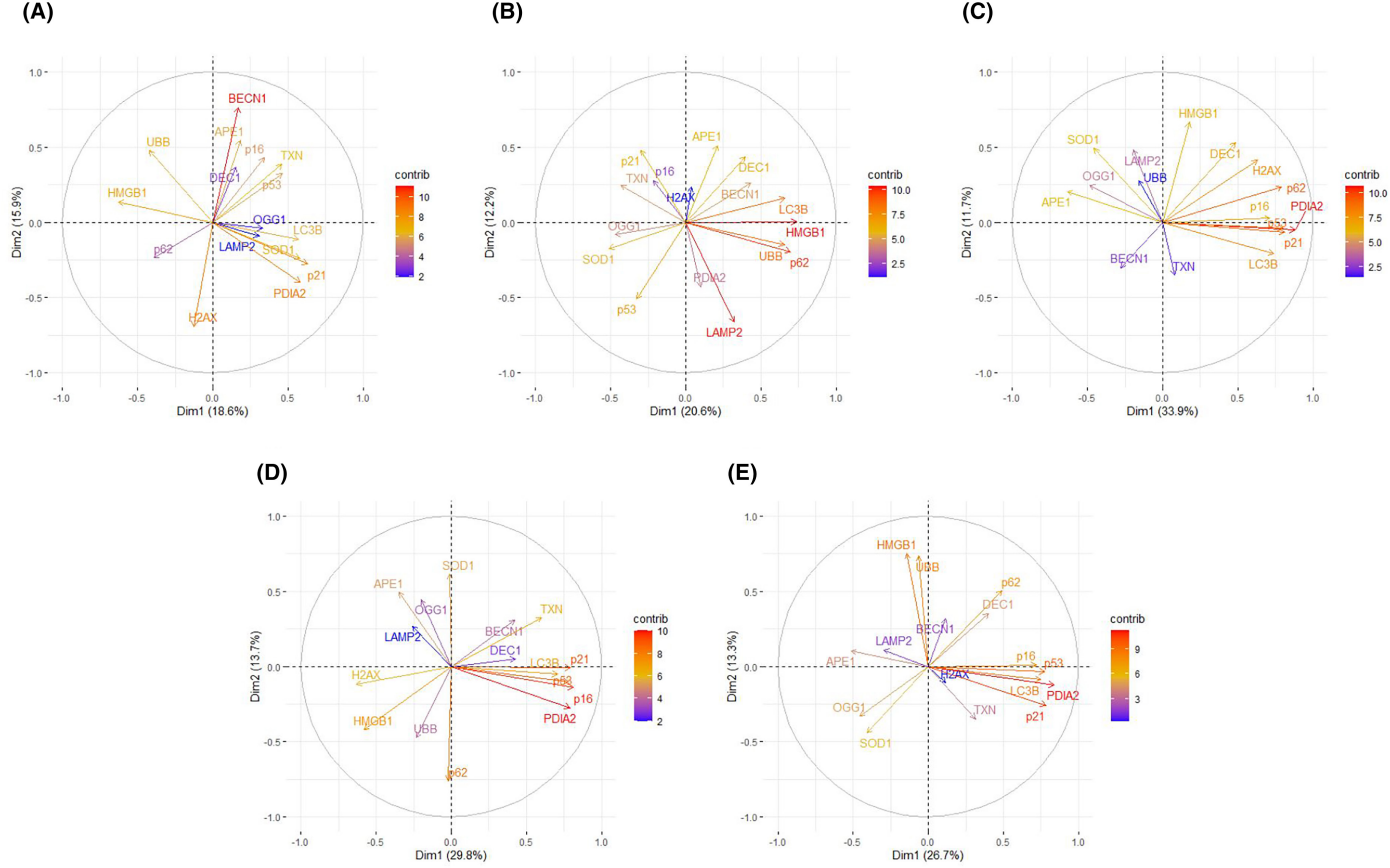
The variable correlation plots of principal component analysis (PCA) on the first two component axes of the expression of genes involved in antioxidant defence, senescence and autophagy in the ageing retina of mice with knockout in the peroxisome proliferator‐activated receptor gamma coactivator 1‐alpha (*PPARGC1A*) gene encoding PGC‐1α gene and wild‐type animals. The contribution of each gene expression level is represented by a colour of a gradient scale (contrib). PCA was performed for 3‐month (A) and 12‐month (B) mice, PGC‐1α knockout (C) and wild‐type (D) mice or data for all kinds of mice (E).

To assess the contribution of specific genes to the total gene expression, a PCA correlation plot was made (Figure 5). The genes whose expression most significantly contributed to the total expression were *BECN1* (autophagy), *HMGB1* (senescence), *PDIA2* (antioxidant defence) and *CDKN2A* (senescence). The contribution of specific genes strongly depended on age and PGC‐1α expression.

The building of the network of interactions on the String Cytoscape basis led to the conclusion that in 3‐month animals PGC‐1α knockout had the most pronounced effect on the expression of the *BECN1* (autophagy, an increase) and *H2AX* (antioxidant defence, a decrease). In 12‐month animals the most pronounced differences between knockout and WT animals were for the *SQSTM1* (autophagy, an increase), *HMGB1* (senescence, an increase), *SOD1* and *TXN* (both antioxidant defence, a decrease). The interaction network showed that, as expected, the greatest number of interactions displayed the product of the *TP53* gene, p53. On the contrary, the product of the *PDIA2* gene directly interacted only with the *TXN* gene product, similarly to the unique interaction of the *BHLHE40* (senescence) with *TP53*.

**FIGURE 6:**
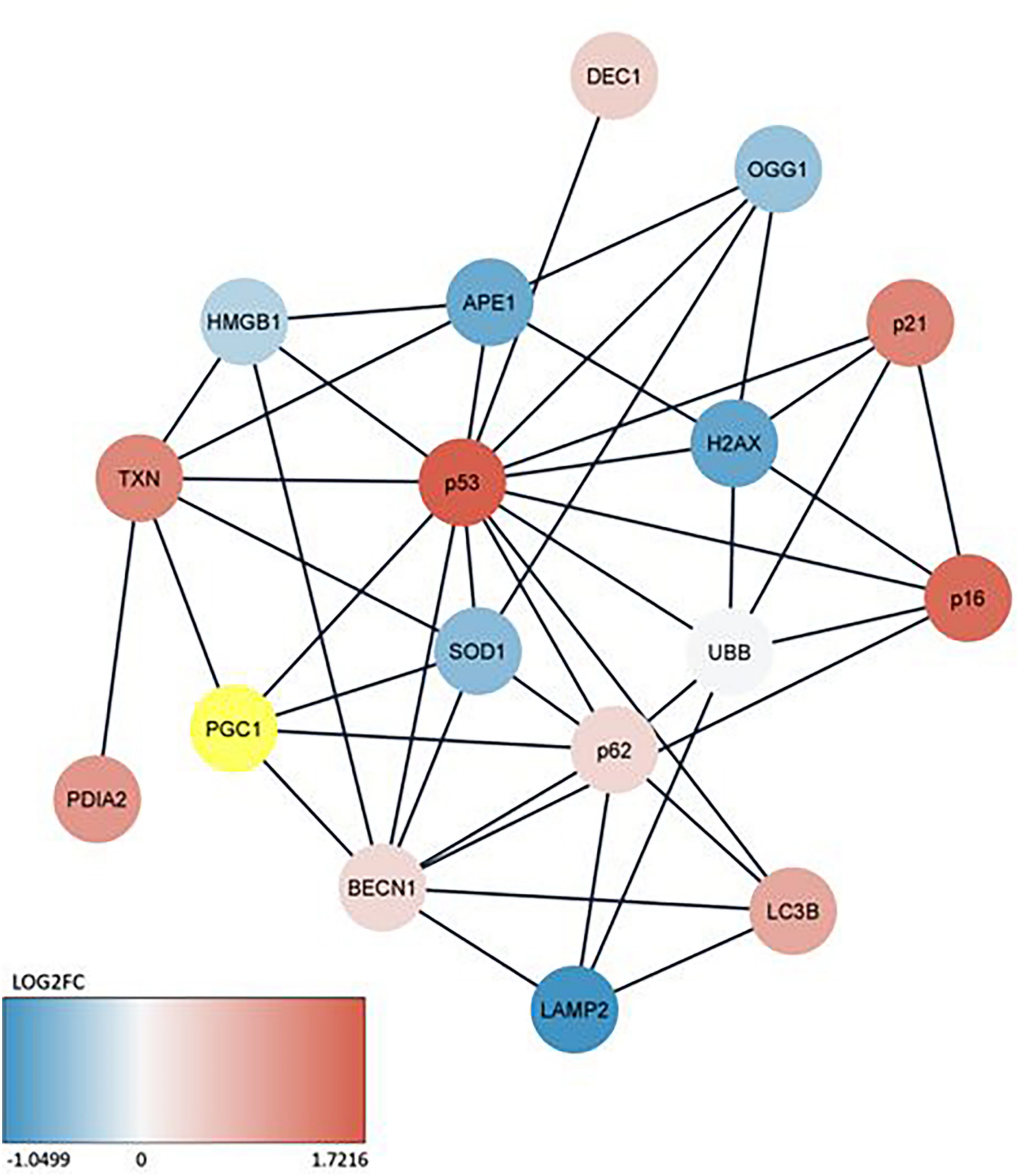
Interaction network for the peroxisome proliferator‐activated receptor gamma coactivator 1‐alpha (*PPARGC1A*) gene encoding the PGC‐1α protein in the mouse ageing retina. The colours of the circles representing specific genes correspond to fold changes expressed by a logarithm of the ratio of the gene expression for 3‐ and 12‐month mice except PGC‐1α represented by a yellow circle and abbreviated to PGC1. The network was built based on String Cytoscape. All abbreviations are defined in the main text.

Finally, we included the *PPARGC1A* in the network of interactions built with String Cytoscape (Figure 6). *PPARCG1A* directly interacted with a substantial number of proteins and only *TP53* had a greater number of interactions.

## 4 DISCUSSION

We used slices of mouse eye sections and determined the expression of markers in situ in RPE cells as, in our opinion, better reflects the reality than studies in the RPE‐derived cell lines. In general, cells in the central part of the retina, including the macula, are quiescent and do not divide due to spatial constraints. They are not post‐mitotic because they can reinitiate cell cycle in vitro, such as ARPE‐19 cells and proliferation may affect the expression of genes.^26^ Therefore, the levels of the markers determined by immunohistochemistry may be more adequate to in vivo situation than immunocytochemistry. However, the question remains, what we determined—gene expression on the protein level or immunohistochemical protein abundance? The next question is why we did not apply a high‐throughput analysis with the use of, for example, microarrays dedicated to antioxidant defence, senescence and autophagy? The answer is the same—mRNA must have been isolated from primary cultures of RPE cells that are difficult to obtain in required quantity and purity and they do not reflect the processes occurring in situ, including gene expression. Analysis data from mRNA expression, including the bioinformatic analysis we performed for proteins, would significantly overload the manuscript with data, making it difficult to read and understand. Such analysis would provide information about mechanism of the expression of the genes involved in senescence, antioxidant defence and autophagy, but it was not the aim of our study. Finally, changes in cellular phenotype and accumulation of inflammatory cells, such as microglia and neutrophils are possible in degenerative processes occurring in RPE during AMD development.^4^, ^27^ Therefore, it may be impossible to isolate RNAs specific to RPE by routine methods, including flash‐freezing.

Results of immunohistochemical studies supported by a deep statistical and bioinformatic analyses suggest that the expression of genes of antioxidant defence, senescence and autophagy depends on age and the presence/absence of the active *PPARGC1* gene encoding the PGC‐1α protein. Moreover, the interplay between these three categories of genes may occur and it can be coordinated by the PGC‐1α protein and depend on age.

We observed an increase in the expression of the p62/SQSTM1 protein, suggesting a reduced autophagy flux, but on the other hand, we noticed an increase in LC3, indicative of an increased autophagy. This apparent inconsistency follows from the dynamic character of autophagy flux, in particular from changes in the concentration of autophagy‐related proteins. We semi‐quantitatively determined the expression of proteins at different stages of autophagy flux. p62/SQSTM1 recognizes ubiquitinated perinuclear protein aggregates and the p62/SQSTM1‐tagged material is then isolated from the cytosol in p62/SQSTM1‐LC3 interaction‐guided autophagosome formation.^27^ In the final step of the autophagy process, a lysosome is fused to the autophagosome resulting in the formation of the autolysosome followed by the degradation of its contents including ubiquitin, p62/SQSTM1 and LC3. Once p62/SQSTM1 is upregulated together with LC3, it indicates relatively decreased autophagy in response to stress as shown in our earlier study.^22^

We showed that ageing might dramatically change the reaction of the retina to the presence and absence of PGC‐1α. The level of SOD1 increased in young KO mice, whereas it decreased in their old counterparts, when considered without interaction between ageing and the presence or absence of PGC‐1α. The main function of SOD1 is converting the superoxide radical into molecular oxygen and hydrogen peroxide, in redox reactions.^28^ Therefore, we can speculate that in young animals a lack of PGC‐1α increased the expression of SOD1, but ageing might attenuate that reaction, finally leading to a decreased level of SOD1 in old animals. Therefore, we can consider that the first reaction of the RPE cells to the lack of PGC‐1α was the increase in the expression of *SOD1* to recompensate its lack, but then during ageing, it decreased. It is important to underline that it was a general picture of the expression of an antioxidant defence protein, not induced by oxidative stress as we expected that the situation may be different in the latter case.

Multivariate testing analysis showed that autophagy and senescence, in contrary to antioxidant defence, discriminated between 3‐month WT and KO animals. The most significantly discriminating proteins in the autophagy group were BECLIN1 encoded by the *BECN1* gene and ubiquitin encoded by the *UBB* gene. BECLIN1 is a component of the class III phosphatidylinositol 3‐kinase (PI3K‐III) complex, which is crucial in the assembly of autophagosomes.^29^ Moreover, it may be important in the regulation of pro‐life and pro‐death functions of autophagy and its connections with apoptosis.^30^ We previously presented arguments that autophagy might regulate RPE cells death in AMD.^31^ Moreover, BECLIN1 is regulated by a cascade of ubiquitination events.^32^ Reversible ubiquitination of essential autophagy inducers is an important mechanism of autophagy regulation.^33^ Moreover, a crosstalk between autophagy and ubiquitin‐proteasome system is crucial for the management of cellular waste, including all‐trans retinal, drusen and lipofuscin, containing unfolded, damaged and no longer needed proteins in RPE cells.^34^ Therefore, ubiquitin, as a cofactor for these processes, may be important in AMD pathogenesis.

The stepwise forward LDA in 3‐ and 12‐month mice demonstrated that the groups of markers we chose distinguished between KO and WT animals. Furthermore, our bioinformatic analysis confirmed an interplay between some genes of antioxidant defence, senescence and autophagy. However, the expression of certain genes was negatively correlated with others. This confirms a complex relationship between the regulation of gene expression and phenotype. Our bioinformatic analysis also showed that p53 had the largest interacting network with other proteins that were studied in this work. It is not very surprised as p53 is involved in antioxidant defence, senescence and autophagy and plays important roles in these effects.^35^, ^36^, ^37^

As we mentioned, the Golestaneh lab showed that PGC‐1α was essential in mitochondrial homeostasis in RPE cells obtained from differentiation of iPSCs obtained from AMD donors.^6^ In this work, we investigated two proteins that were directly related to mitochondrial quality control (mtQC): PINK1 and PRKN. These proteins, along with optineurin (OPT), belong to the main proteins of the mitophagy pathway.^38^ In normal conditions, PINK1 is imported into and degraded inside mitochondria, but when a damage occurs, PINK1 is accumulated on the outer mitochondrial membrane and PRKN is recruited from the cytosol.^39^ Therefore, the PINK1/PRKN ratio is a good indicator of damaged mitochondria and mitophagy. We did not observe any influence of PGC‐1α on the level of PINK1/PRKN in young mice, but this ratio increased in old KO animals, indicating that the lack of PGC‐1α might result in mitochondrial damage (Figure 5). Dysfunctional mitochondria and impaired mitophagy in RPE cells are important elements in AMD pathogenesis.^40^ However, we investigated other proteins than PINK1 and PRKN that might be associated with mitochondrial maintenance in RPE cells as antioxidant defence and senescence interwind in mtQC and along with inflammation may contribute to neurodegeneration.^41^, ^42^

It was shown that different isoforms of vascular endothelial growth factor (VEGF), a molecule that is critical for choroidal neovascularization and wet AMD, are differentially expressed during mouse development and in its adulthood.^43^ However, PGC‐1α can be, at least in part, replaced by its analogue, PGC‐1β, so apart from PGC‐1α knockout, PGC‐1β and PGC‐1α/PGC‐1β knockouts should also be used to completely assess the role of the PGC‐1 family in AMD pathogenesis.^44^

Our study is not highly innovative, but we cannot find other results integrating senescence, antioxidant defence and autophagy in an age‐dependent AMD model. Moreover, we cannot find such deep bioinformatic analysis of the joint expression of genes related to these three categories.

In summary, PGC‐1α may be involved in the interplay between antioxidant defence, senescence, and autophagy, important in AMD pathogenesis and ageing may strongly modulate this interplay. Mice with knockout in the *PPARGC1A* gene may be suitable to investigate early and late signs of PGC‐1α‐related effects in AMD pathogenesis and therefore provide model for novel drug development.

## AUTHOR CONTRIBUTIONS

**Iswariyaraja Sridevi Gurubaran:** Data curation (equal); writing – review and editing (equal). **Cezary Watala:** Data curation (equal); formal analysis (lead); investigation (equal); writing – review and editing (equal). **Joanna Kostanek:** Formal analysis (supporting); investigation (equal); methodology (equal); writing – review and editing (equal). **Joanna Szczepanska:** Investigation (equal); writing – review and editing (equal). **Elzbieta Pawlowska:** Investigation (equal); writing – review and editing (equal). **Kai Kaarniranta:** Conceptualization (equal); writing – review and editing (equal). **Janusz Blasiak:** Conceptualization (equal); supervision (equal); writing – original draft (equal); writing – review and editing (equal).

## CONFLICT OF INTEREST STATEMENT

The authors declare no conflict of interest of any other conflict associated with this manuscript.

## Data Availability

The data that support the findings of this study are available from the corresponding author upon reasonable request.
